# The effect of risedronate on osteogenic lineage is mediated by cyclooxygenase-2 gene upregulation

**DOI:** 10.1186/ar3122

**Published:** 2010-08-25

**Authors:** Maria Teresa Valenti, Sandro Giannini, Luca Donatelli, Mirko Zanatta, Francesco Bertoldo, Stefania Sella, Maria Teresa Vilei, Elena Ossi, Giuseppe Realdi, Vincenzo Lo Cascio, Luca Dalle Carbonare

**Affiliations:** 1 Clinic of Internal Medicine D, Department of Medicine - University of Verona Piazzale L. Scuro, 10 - 37134 Verona, Italy; 2 1st Medical Clinic, Department of Medical and Surgical Sciences, University of Padova - Via Giustiniani, 2 - 35128 Padova, Italy

## Abstract

**Introduction:**

The purpose of this study was to evaluate the effects of risedronate (Ris) in the modulation of bone formation in rats with glucocorticoid (GC)-induced osteoporosis by histomorphometric, immunohistochemical and gene expression analyses.

**Methods:**

We analyzed structure, turnover and microarchitecture, cyclooxygenase 2 (COX-2) levels and osteocyte apoptosis in 40 female rats divided as follows: 1) vehicle of methylprednisolone (vGC) + vehicle of risedronate (vRis); 2) Ris 5 μg/Kg + vGC; 3) methylprednisolone (GC) 7 mg/Kg + vRis; 4) GC 7 mg/Kg +Ris 5 μg/Kg. In addition, we evaluated cell proliferation and expression of COX-2 and bone alkaline phosphatase (b-ALP) genes in bone marrow cells and MLO-y4 osteocytes treated with Ris alone or in co-treatment with the selective COX-2 inhibitor NS-398 or with dexametasone.

**Results:**

Ris reduced apoptosis induced by GC of osteocytes (41% vs 86%, *P *< 0.0001) and increased COX-2 expression with respect to controls (Immuno-Hystochemical Score (IHS): 8.75 vs 1.00, *P *< 0.0001). These positive effects of Ris in bone formation were confirmed by *in vitro *data as the viability and expression of b-ALP gene in bone marrow cells resulted increased in a dose dependent manner.

**Conclusions:**

These findings suggest a positive effect of Ris in bone formation and support the hypothesis that the up-regulation of COX-2 could be an additional mechanism of anabolic effect of Ris.

## Introduction

Bisphosphonates (BPs), synthetic analogs of pyrophosphate, are the most effective inhibitors of bone resorption and are currently used in the treatment of several bone diseases. Their mechanism of action has been well described. They bind tightly to bone mineral surface, penetrate into osteoclasts and stimulate their apoptosis through the inhibition of the mevalonate pathway [1].

Recent findings suggest that bisphosphonates may also indirectly suppress bone resorption through their action on osteoblasts [2], and osteocytes [3], which could represent another target for these drugs.

Instead, it is not clear if BPs have also a beneficial influence on the bone formation process. Histomorphometric analysis in osteoporotic subjects indicates that BPs may increase the mean wall thickness and reduce the imbalance between formation and resorption at the basic multicellular unit (BMU) [4,5], leading to a continuing increase in bone mineral density (BMD) even after a long period of treatment, as demonstrated in clinical studies [6].

They also control osteoblastic proliferation and differentiation [7,8], modulate osteoblast production of extracellular matrix proteins, regulate the secretion of several cytokines and growth factors [9,10] and enhance proliferation and maturation of bone marrow stromal cells to osteoblastic lineage [11]. Bisphosphonates are also able to prevent apoptosis of osteoblasts and osteocytes induced by glucocorticoid therapy [12]. It is well known that osteocytes are well differentiated osteoblasts regularly spaced throughout the mineralized matrix. They are believed to detect bone microdamage and to transmit signals leading to its repair [13,14].

The disruption of the osteocyte network could compromise this mechanism, leading to accumulated microdamage and increased bone fragility. Such a defect in bone quality could account for the higher incidence of fractures and the disproportion between the significant increase in bone fragility and the relative small decrease of BMD observed, for example, in glucocorticoid induced osteoporosis [12,15].

In our previous histomorphometric study on glucocorticoid-induced osteoporosis [16] we found that rats treated with risedronate (Ris) showed increased trabecular number and thickness and decreased trabecular separation not only with respect to glucocorticoid treated rats but also with respect to controls. In addition, increased wall thickness, the end product of osteoblastic activity, was observed. In terms of turnover, rats treated with Ris showed a reduced activation frequency with an increased active Formation Period. All these findings suggest an effect of Ris on osteoblastic lineage and support the hypothesis of a neoformative activity of bisphosphonates.

The mechanism by which bisphosphonates stimulate osteoblasts is not yet completely elucidated. Giuliani *et al. *[10] reported that the anabolic effect of BPs was associated with the stimulation of b-FGF, while Mundy *et al. *demonstrated that the anabolic effect of statins, which influence the mevalonate pathway as BPs, was due to their stimulation of Bone Morphogenetic Protein-2 (BMP-2) [17]; BMP-2 gene expression was also upregulated during osteoblast maturation after BP treatment [18]. Von Knoch *et al. *showed that a cascade of osteoblast-related genes including *BMP-2, cbfa-1, type 1 collagen *and *Bone Sialo-Proteins *(*BSP*) were up regulated and significantly increased in bone marrow stromal cells after BP treatment [11]. The Osteoprotegerin/Receptor Activator of Nuclear Factor-κB Ligand (OPG/RANKL) system is also influenced by BPs and may be related, at least in part, to the stimulatory effects of BPs on osteoblastic differentiation [2].

In addition, it has been shown that exogenous and endogenous prostaglandins (PGs) modulate both bone formation and resorption [19-23]. PGs are produced by cyclooxygenase (COX), which is a rate-limiting enzyme that converts arachidonic acid to PGs [24]. Three isoforms of COX are recognized: COX 1, which is constitutively expressed, COX-2, which is inducible by multiple factors and involved in PGs production during inflammation and other acute responses [25] and COX-3, which has recently been related to paracetamol-induced hypothermia and analgesia [26,27]. On the contrary, the inhibition of cyclooxygenase (COX) has been associated with decreased bone formation *in vivo *[28] and delayed experimental fracture healing [29].

Strontium ranelate, a new treatment for postmenopausal osteoporosis that exerts both antiresorptive and anabolic effects on bone, is able to influence prostaglandins metabolism. Strontium ranelate induces COX-2 expression and promotes PGE2 production and activity in murine primary calvarian osteoblast trough ERK pathway [30]. It also increases osteoblastic differentiation and mineralization, acting on early osteoblastic precursors to induce COX-2 and PGE2 production [31].

Up to now, studies about the effects of BPs on cyclooxygenase are lacking. On the basis of these data, we hypothesized that BPs could upregulate COX-2 expression according to their putative anabolic effect on bone previously suggested by histomorphometric results. To verify this hypothesis, we studied the effect of Ris by evaluating histomorphometric parameters in rat tibiae and apoptosis and COX-2 expression in femur specimens in the presence or absence of a negative effect on osteoblastic/osteocytic lineage induced by glucocorticoids. To verify the presence of a direct stimulus of Ris on osteoblastic cells, we analysed *in vitro *the viability and *COX-2 *gene expression in bone marrow stromal cells treated with Ris at different concentrations, in the presence and absence of NS-398, a selective COX-2 inhibitor [32]. In addition, to analyze the effects of bisphosphonates on osteogenic differentiation, we evaluated the mRNA expression of *bone marker Alkaline Phosphatase *(*b-ALP*) on stromal cells treated with Ris at different concentrations. On the same model, we investigated a possible relationship between COX-2 pathway and osteogenic differentiation by analyzing mRNA *b-ALP *expression in presence or absence of COX-2 inhibitor.

## Materials and methods

### Animals

Forty six-month-old female Sprague-Dawley rats of approximately 200 to 300 g body weight were obtained from Charles River Italia (Calco, Italy). All rats were housed under similar conditions. They were fed a standard rodent diet containing 0.97% calcium, 0.85% phosphorus, 1,045 IU/Kg vitamin D3, 22.5% protein, 5.5% fat, 52% carbohydrate, and were given access to tap water *ad libitum*.

The animal procedures were approved by the local government authorities and were conducted in accord with accepted standards of humane animal care, as outlined in the Ethical Guidelines.

### Drugs

Risedronate (Ris) in powder form was provided by Procter and Gamble Pharmaceuticals Inc. (Cincinnati, OH, USA). Ris was dissolved in deionized water and was administered at 5 μg/Kg subcutaneously (s.c.) three times a week. Methylprednisolone (GC, Solu-Medrol - Pharmacia, Stockholm, Sweden)) was diluted in a sesame oil vehicle at a concentration of 7 mg/kg.

### Study protocol *in vivo*

At the beginning of the study, rats were randomly divided into four groups (10 rats each) and treated s.c. three times a week as follows:

1. Control group: GC vehicle (vGC) + Ris vehicle (vRis);

2. Ris group: Ris 5 μg/Kg body weight + vGC;

3. GC Group: GC 7 mg/Kg + vRis;

4. GC + Ris Group: GC 7 mg/Kg and Ris 5 μg/Kg.

The rats were weighed once a week to adjust the drug dose to body weight. The study's experimental period lasted 30 days. To evaluate dynamic parameters of bone formation, the rats received double fluorochrome labeling with demeclocycline, 25 mg/Kg s.c. on Day -14 and -13, and with calcein, 10 mg/Kg s.c. on Day -4 and -3 prior to sacrifice.

### Histomorphometry

The right tibiae were removed, dissected free of soft tissue, and fixed in 70% reagent alcohol. The samples were embedded undecalcified in methyl-methacrylate resin (Merck 800590, Darmstadt, Germany).

Measurements were performed by means of an image analysis system consisting of an epifluorescent microscope (Leica DMR, Leica Microsystems, Wetzlar, Germany) connected to an analogic 3 CCD camera (Sony DXC 390P; Sony, Tokio, Japan) and a computer equipped with a specific software for histomorphometric analyses (Bone, Explora Nova, La Rochelle, France). The area analyzed was restricted to the trabecular bone of the secondary spongiosa area between 2 and 4 mm distal to the growth plate-metaphyseal junction [33].

Histomorphometric parameters are reported in accordance with the ASBMR Committee nomenclature [34].

Thickness results were adjusted for the obliquity of sections by multiplying by π/4 [34].

### Immunohistochemical determination of COX-2 expression

Explants of left femora were fixed in 4% buffer formalin for 24 hours, decalcified with 0.5 mol/L ethylendiaminetetraacetic acid, pH 8, for 7 to 10 days, paraffin embedded, and cross sectioned (5 μm thick) at three different levels. Goat Serum was purchased from Sigma-Aldrich Co. (St. Louis, MO, USA); Primary Antibody, rabbit anti-mouse cox-2, from NeoMarkers Inc. (Fremont, CA, USA); Secondary Antibody, goat anti-rabbit IgG-B, from SantaCruz Biotechnology Inc., Santa Cruz, CA, USA; ABC solution, Vectastain ABC kit, from Vector Laboratories Inc. (Burlingame, CA, USA); DAB solution, Liquid DAB Substrate Chromogen System, from DakoCytomation (Fort Collins, Colorado, USA). Immunohystochemistry was performed on paraffin-embebbed specimen sections, following the technique of Fortier *et al. *[35], with the same modification. Briefly, slides were stored in the stove at 60°C till paraffin loosed, and then were washed in xylene, followed by rehydration through graded ethanol washes. Permeabilization was performed by heating at 70° in a humidified chamber, the sections were previously covered with 10 mM citrate buffer, pH 6.0, followed by cooling at room temperature. The endogenous peroxidases were blocked by incubation in DI water 0.3% H2O2, and non-specific binding was prevented by incubation in PBS 10% Goat Serum. The first antibody (rabbit anti-mouse COX-2) was diluted 1:25 in PBS 10% Goat Serum, and the slides were incubated overnight at 4°C in humidified chamber. Slides were then washed in 1% Triton PBS, following a second blocking step with PBS 10% Goat Serum. Secondary biotinylated antibody (goat anti-rabbit IgG-B) was added at the dilution of 1:100. After 30 minutes of incubation at room temperature, sections were washed in PBS 1% Triton X-100 followed by only PBS, before staining by use of a Vectastain peroxidise standard ABC kit, and DAB solution, both according to the suppliers' protocol. Cells were then counterstained with haematoxylin and mounted with glycerol solution. For each sample, six random fields at 40 × of magnification were analyzed by counting positive cells with respect to total cells. Results were expressed as an immunohistochemical score (IHS), based on the German Immunoreactive Score, which combines quantity and intensity values. The IHS is calculated by combining the percentage of immunoreactive cells (quantity score, %) with an estimate of the staining intensity; quantity score: no staining is scored as 0; 1 to 10% of cells stained scored as 1; 11 to 50% as 2; 51 to 80% as 3; and 81 to 100% as 4; staining intensity was rated on a scale of from 0 to 3, with 0 being negative, 1 weak, 2 moderate, and 3 strong. The raw data were converted to IHS by multiplying the quantity and staining intensive score.

### Apoptosis

Apoptotic nuclei in paraffined tissue were identified by Terminal deoxynucleotidyl Transferase-Biotin-dUTP nick end labeling (TUNEL) technique. The procedure was the one reported in the manufacturer's instruction (Roche, Mannheim, Germany). Apoptotic nuclei were identified by the red precipitate obtained by incubating the glass slides with 0.04% 3-amino-9-ethyl- carbazole (AEC) in 50 mM sodium citrate buffer, pH 5 containing 0.015% H_2_O_2_. The values were expressed as the percentage of TUNEL-position nuclei with respect to the total haematoxylin-stained nuclei. Six different fields with about 40 total cells for each sample were measured.

### Study protocol *in vitro*

#### Cell culture

β-glycero-phosphate, 0.1 mM L-ascorbic 2-phosphate and dexametasone were purchased from StemCell (StemCell Technologies, Vancouver, BC, Canada). Almost all other chemicals were from Sigma (St Louis, MO, USA). Other companies are specified in the text.

Bone marrow cells were obtained from 8 to 10 week-old Sprague-Dawley rats. The femurs were removed aseptically and dissected, the ends of bones were cut and the marrow was flushed out with DMEM by using a needle and syringe. The marrow was disperded gently by pipetting several times to obtain a single cell suspension and the cells were counted with a hemocytometer. Cells were seeded into 25 ml cell culture flasks at a density of 1 × 10^6 ^cells/ml and culture for 48 h at 37°C in a humidified atmosphere with 5% CO_2 _in DMEM containing 2 mM L glutamine, 20% fetal bovine serum, 100 U/ml streptomycin and 100 μg/ml penicillin. After 48 h, all nonadherent cells were removed and the adherent cells were grown for an additional period. At sub-confluence, cells were detached with trypsin and at the third passage the cells were plated in a 96-well cell culture plate or 25 ml cell culture flasks at a density of 3.0 × 10^5 ^cells/ml. The cells were cultured in osteogenic differentiation medium consisting of DMEM with 10% FBS supplemented with 10 mM β-glycero-phosphate and 0.1 mM L-ascorbic 2-phosphate.

MLO-Y4 osteocytes, a cell line derived from mouse long bones (provided by Dr. L. F. Bonewald, University of Missouri, Kansas City, MO [36]), was cultured in -minimum essential medium (-MEM) supplemented with 2.5% fetal bovine serum (FBS; Gibco, Grand Island, NY, USA), 2.5% calf serum (Gibco), 100 U/ml streptomycin and 100 μg/ml penicillin at 37°C and 5% CO_2 _in air. The cells were cultured in osteogenic differentiation medium consisting of -MEM with 2.5% fetal bovine serum, 2.5% calf serum, 100 U/ml streptomycin and 100 μg/ml penicillin supplemented with 10 mM β-glycero-phosphate and 0.1 mM L-ascorbic 2-phosphate.

The cultures were treated with Ris at concentrations ranging from 0.1 to 10 μM with or without NS-398 (0.1 μM), or with dexametasone (1 μM) as specified in the figures, and controls were treated with vehicle (DMSO, < 0.1%).

#### XTT assay

Cell viability was evaluated by a colorimetric assay based on the reduction of the tetrazolium salt XTT [(sodium 3^I^-(1-phenylamino-carbonyl-3,4-tetrazolium)-bis(4-methoxy-6-nitro)] benzene sulfonic acid hydrate) by mitochondrial dehydrogenase of viable cells to a formazan dye (Cell proliferation kit II -- XTT Roche). Briefly, cells were cultivated in 96 microtitre plates in medium containing different concentrations of Ris with or without NS-398. After 72 h, 50 μl XTT labeling mixture was added to each well and incubated at 37°C for 4 h. The spectrophotometric absorbance of the samples was measured using a microtitre plate (ELISA) reader at a wavelength of 450 nm.

#### Total RNA extraction and reverse transcription

Total RNA was extracted from each cell pellet using the RNAeasy minikit (Qiagen, Duesseldorf, Germany) with DNAse I treatment. The amount and purity of the RNA were checked by measuring the absorbance at 260 and 280 nm, and where a ratio ranging from 1.8 to 2.0 was taken to be pure. First-strand cDNA was generated from 1 μg of total RNA using the First Strand cDNA Synthesis Kit (GE Healthcare, Buckinghamshire, United Kingdom), with random hexamers, (GE Healthcare) according to the manufacturer's protocol. RT product was aliquoted in equal volumes and stored at -80°C.

#### Real time RT-PCR

mRNA quantification was analyzed by Relative Standard Curve Method (Chemistry guide Applied Biosystems (Foster City, CA, USA). PCR was performed in a total volume of 25 μl containing 1 × Taqman Universal PCR Master mix, no AmpErase UNG and 5 μl of cDNA from each sample; pre-designed, Gene-specific primers and probe sets for each gene were obtained from Assay-on-Demande Gene Expression Products (Rat (RN01483828-m1, *Cox2*; RN01516028-m1, *b-ALP*; RN00667869-m1, *ACTb*) (mouse (Mm01307329-m1, *Cox2*; Mm01187117-m1, *b-ALP*; *ACTb *human) Applied Biosystems). The Real Time amplifications included 10 minutes at 95°C (AmpliTaq Gold activation), followed by 40 cycles at 95°C for 15 seconds and at 60°C for 1 minute. Thermocycling and signal detection were performed with ABI Prism 7300 Sequence Detector (Applied Biosystems). Signals were detected according to the manufacturer's instructions and we selected the ΔRn in the exponential phase of amplification plots to determine the Ct values and to obtain the linearity of calibration curves.

PCR efficiencies were calculated with a relative standard curve derived from a four cDNA dilution series in triplicate and gave regression coefficients greater than 0.98 and efficiencies greater than 96%. To normalize gene expression we amplified the housekeeping gene *ACTb*. The gene expression levels were calculated for each sample in triplicate after normalization against the housekeeping gene (*ACTb*), using the relative fold expression differences [37].

### Statistical analysis

Results were expressed as means ± SD. The statistical analysis was assessed by one way and two way analysis of variance (ANOVA). In all analyses, a *P-*value less than 0.05 (*P *< 0.05) was considered a significant difference. Differences among groups yielding a statistical significance with *P *< 0.05 were tested with Bonferroni as a *post-hoc *test. Statistical analyses were performed by using SPSS for Windows version 16.0 (SPSS Inc., Chicago, IL, USA).

## Results

### *In vivo* study

#### Histomorphometric effects of risedronate

BV/TV was higher in Ris treated groups with respect to GC and control groups. Accordingly, Ris-treated group showed higher Trabecular Thickness and Number and lower Trabecular Separation than GC and control groups (Table 1). In addition, Ris treated groups showed increased Wall Thickness, the end product of osteoblastic activity. As for turnover, we observed decreased Activation Frequency in Ris treated rats, confirming our previous findings [16].

**Table 1:** Histomorphometric results in rats treated with glucocorticoids (GC) and risedronate (Ris)

	Controls	Ris	GC	GC+Ris
Bone volume/tissue volume (%)	35 ± 1	39 ± 1(b,c)	31 ± 1(b)	36 ± 1(a)
Trabecular thickness (μm)	50 ± 1	55 ± 1(b)	43 ± 1(b)	53 ± 1(a)
Trabecular number (N/mm)	3.4 ± 0.2	4.6 ± 0.1(b)	2.9 ± 0.1(b)	4.4 ± 0.1(a,b)
Trabecular separation (μm)	155 ± 6.5	139 ± 4.8(b,c)	273 ± 5.3(b)	144 ± 6.1(a)
Mineral apposition rate (μm/day)	0.66 ± 0.04	0.65 ± 0.04	0.62 ± 0.05(b)	0.65 ± 0.04
Mineralized surface/BS (%)	11.2 ± 1.5	9.8 ± 0.9(b)	9.6 ± 0.9(b)	9.8 ± 1.0(b)
Bone formation rate/BS (μm^3^/μm^2^/day	7.4 ± 1.2	6.3 ± 0.6(b)	5.9 ± 0.6(b)	6.3 ± 0.6(b)
Wall thickness (μm)	16.8 ± 1.3	24.9 ± 3.6(b)	12.0 ± 3.1(b)	22.8 ± 5.9(a,b)
Activation frequency (N/yr)	1.6 ± 0.3	0.9 ± 0.2(b)	1.9 ± 0.6	1.0 ± 0.3(a,b)
Active formation period (days)	25.4 ± 2.0	38.8 ± 5.4(b)	19.4 ± 4.8	35.3 ± 9.3(a,b)
Eroded surface/BS (%)	1.9 ± 1.0	1.7 ± 0.7	2.2 ± 1.1	1.9 ± 1.1

BS, bone surface.

a) *P *< 0.001 vs GC. b) *P *< 0.05/*P *< 0.001 vs controls. c) *P *< 0.001 vs GC + Ris.

All values are expressed as mean ± SD.

#### Effects of risedronate and glucocorticoids on osteocytic apoptosis

**Figure 1:**
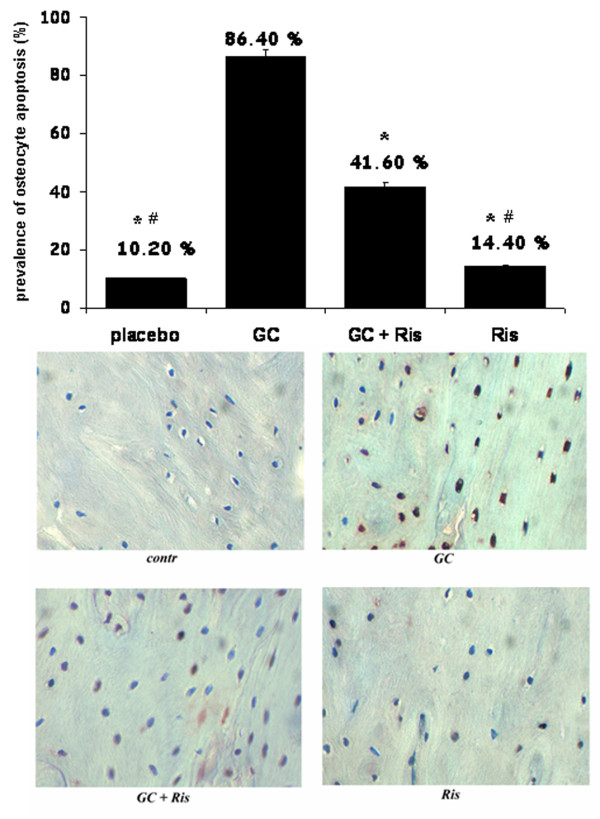
**Osteocyte apoptosis (TUNEL) in rats treated with risedronate (Ris) and glucocorticoids (GC)**. TUNEL positive apoptotic cells with respect to total cells expressed as percentage were quantified in randomly selected six microscopic fields (200 μm square, each) under 40 × magnification. * *P *< 0.001 vs GC; # *P *< 0.001 vs GC +Ris.

According to Plotkin *et al. *[12], rats treated with GC showed a significant increase of apoptosis with respect to controls (86.4 vs 10.2%, *P *< 0.001, Figure 1), reduced by the addition of Ris.

#### Effects of risedronate and glucocorticoids on osteocytic COX-2 expression

**Figure 2:**
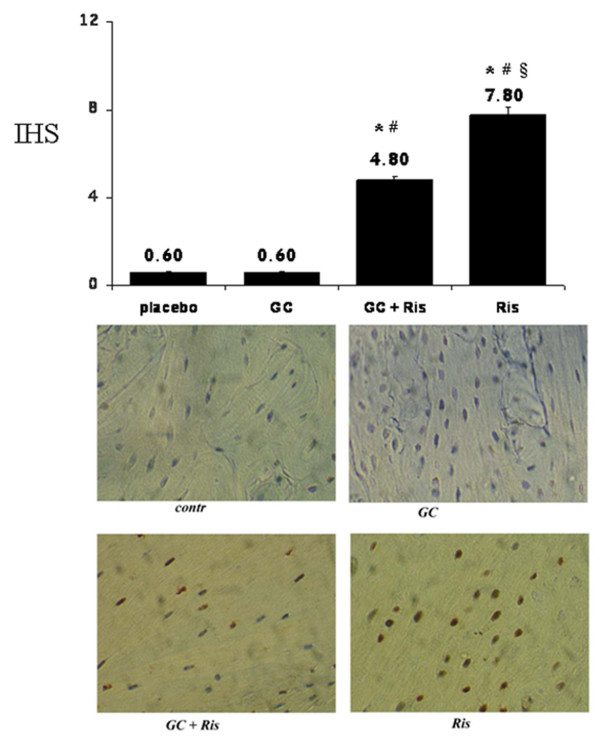
**COX-2 expression in osteocytes of treated rats. COX-2 positive osteocytes in rats treated with risedronate (Ris) and glucocorticoids (GC)**. Results were expressed as Immuno-Hystochemical Score, IHS), based on German Immunoreactive. * *P *< 0.001 vs controls; # *P *< 0.001 vs GC; § *P *< 0.001 vs GC+Ris.

We evaluated COX-2 expression in all rats. Rats treated with Ris showed an increased expression of osteocytic COX-2 vs placebo group (IHS: 7.8 vs 0.6, *P *< 0.001, Figure 2). On the contrary, rats treated with GC did not show any significant difference in COX-2 expression with respect to placebo.

On the other hand, the combined treatment (GC and Ris) induced a significant increase of COX-2 expression with respect to the placebo (IHS: 4.8 vs 0.6, *P *< 0.001), even if significantly lower than Ris alone treated group (IHS: 4.8 vs 7.8, *P *< 0.001). Furthermore, increased COX-2 gene expression observed with Ris was maintained even in the presence of GC, suggesting that Ris counteracts negative effects of GC on osteoblastic lineage.

### *In vitro* study

#### Effects of risedronate on cell viability

**Figure 3:**
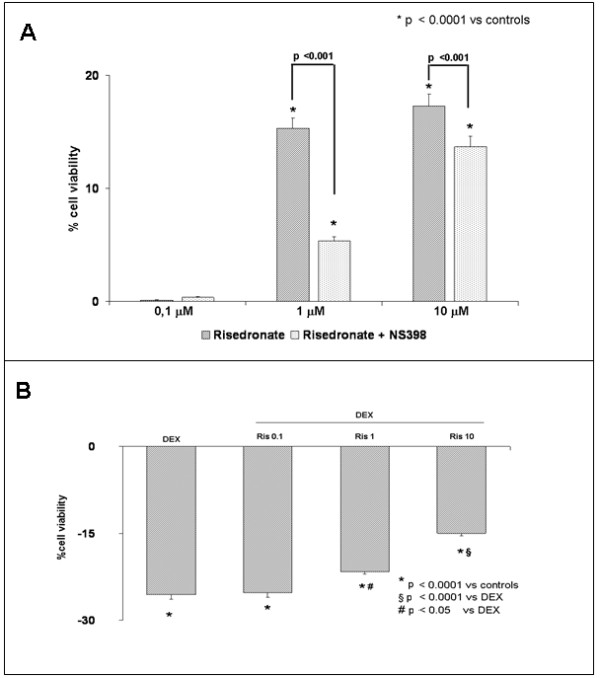
**Viability values in bone marrow stromal cells**. Difference of viability (XTT test) with respect to controls expressed in percentage in bone marrow stromal cells treated with risedronate (Ris) at different concentrations, with or without NS-398 COX-2 inhibitor **(a) **and with dexametasone 1 μM (DEX) **(b) **with or without risedronate (Ris) at different concentrations. The experiments were performed in triplicate.

**Figure 4:**
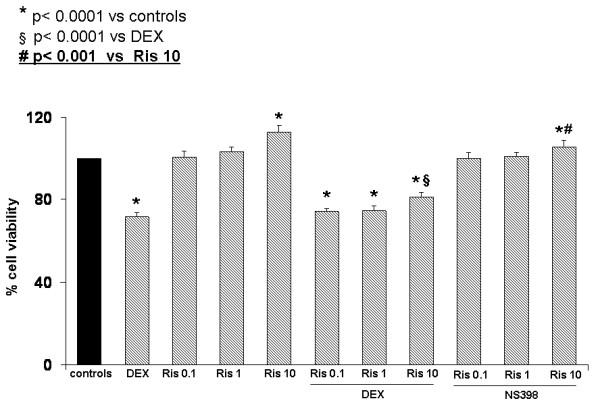
**Viability values in MLO-y4 osteocyte cell line**. Difference of viability (XTT test) expressed in percentage in MLO-y4 osteocytes treated with dexametasone 1 μM (DEX), with or without risedronate (Ris) at different concentrations. In the right side of the figure are also reported the effects on viability of risedronate (Ris) at different concentrations with or without NS-398 COX-2 inhibitor. The addition of NS-398 limited cell viability increased by Ris. This effect was significant at the highest concentration of Ris. The experiments were performed in triplicate.

We evaluated, using bone marrow stromal cells and MLO-y4 osteocytes, the effect of Ris at concentrations of 0, 0.1 to 10 μM with or without COX-2 inhibitor NS-398 (0.1 μM) after three days of culture. The increased levels of cell viability obtained by XTT test at different concentrations of Ris are reported in Figure 3a. This effect could be associated with an increased proliferation and/or survival of cells. Nevertheless, when we inhibited the COX-2 pathway using NS-398, the effect of Ris on cell viability was significantly reduced, at least at the highest concentrations. We also evaluated cell viability in bone marrow stromal cells and MLO-y4 osteocytes treated with dexametasone with or without risedronate. Our results in bone marrow stromal cells (Figure 3b) showed that Ris reduces the negative effect on viability induced by dexametasone in a dose dependent manner. In addition, we observed similar results in the MLO-y4 osteocytes (Figure 4). Also in MLO-y4, the addition of NS-398 reduced the cell viability increased by Ris. This effect was significant at the highest concentration of Ris (Figure 4).

#### Effects of risedronate on gene expression

**Figure 5:**
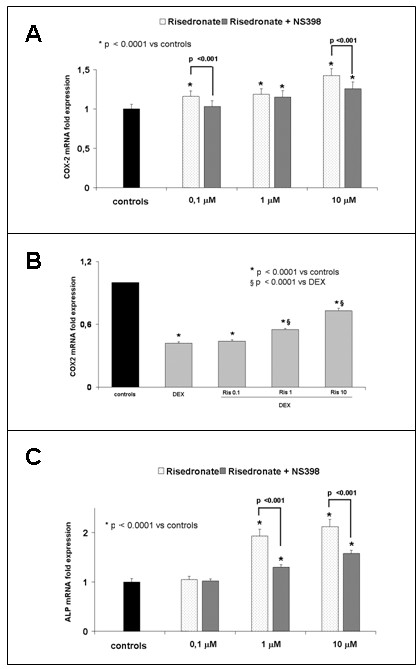
***COX-2 *and *b-ALP *gene expression in treated bone stromal cells**. **(a)***COX-2 *gene expression of bone marrow stromal cells treated with risedronate (Ris) at different concentrations (0.1 to 10 μM) in presence or absence of NS-398 COX-2 inhibitor. Note that Ris induces significant *COX-2 *expression in a dose dependent manner. **(b)***COX-2 *gene expression of bone marrow stromal cells treated with risedronate (Ris) at different concentrations (0.1 to 10 μM) in presence of dexametasone 1 μM (DEX). **(c)***bALP *gene expression of bone marrow stromal cells treated with risedronate (Ris) at different concentrations in presence or absence of NS-398 COX-2 inhibitor. The experiments were performed in triplicate.

RT Real Time PCR analysis was carried out to assess the expression of *COX-2 *and bone marker Alkaline Phosphatase (*b-ALP*) genes in bone marrow stromal cells and MLO-y4 osteocytes. In bone marrow stromal cells, after 72 h of Ris treatment, the expression of genes coding for *COX-2 *and *b-ALP *were upregulated in a dose dependent manner (Figure 5a, c). To test the effect of *COX-2 *in terms of response to Ris treatment, the cultures were treated with or without the NS-398. We observed a significant decrease in *b-ALP *gene expression (and obviously in *COX-2 *gene expression) in cells treated with NS-398, confirming a reduced recruitment of stromal cells to osteoblastic lineage.

**Figure 6:**
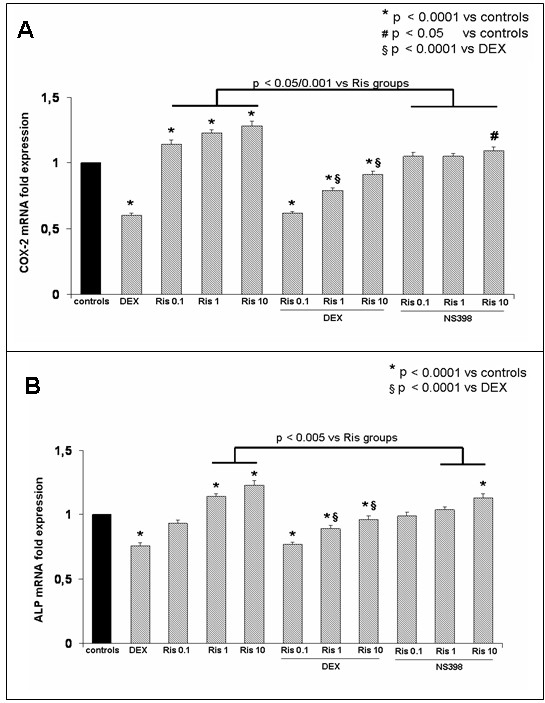
***COX-2 *and *b-ALP *gene expression in treated MLO-y4 osteocyte cell line**. *COX-2 ***(a) **and *b-ALP ***(b) **gene expression in MLO-y4 osteocytes treated with dexametasone 1 μM (DEX), with or without risedronate (Ris) at different concentrations. In the right side of the figure are also reported the effects on gene expression of risedronate (Ris) at different concentrations with or without NS-398 COX-2 inhibitor. Risedronate upregulated the gene expression in a dose dependant manner and reduced the downregulation of gene expression induced by dexametasone. The addition of NS-398 inhibitor significantly reduced the expression of *COX-2 *(*P *< 0.05/0.001) and *b-ALP *(*P *< 0.005) increased by Ris. The experiments were performed in triplicate.

In addition, we evaluated the effect of glucocorticoid on *COX-2 *expression in response to Ris. We found that Ris reduced the downregulation due to DEX in a dose dependent manner (Figure 5b). The same results we obtained in MLO-y4 osteocytes, after 72 h of Ris treatment (Figure 6a, b). The addition of NS-398 inhibitor decreased significantly the Ris-induced *COX2 *and *b-ALP *gene overexpression.

## Discussion

Clinical trials highlighted that patients with postmenopausal osteoporosis treated long term with bisphosphonates show a continuous increase of bone density, an effect that might not be explained simply by the contraction of the remodeling space expected from the inhibition of bone resorption [6,38-40].

Our histomorphometric data show that the main effect of Ris on remodeling in glucocorticoid-induced osteoporosis is the prolonged lifespan of osteocytes, characterized by reduced Bone Formation Rate, activation frequency and prolonged active Formation Period associated with increased wall thickness, according to our previous results [16]. The inhibition of remodelling along with the increase of wall thickness supported the hypothesis of a direct effect of Ris on osteoblastic lineage.

Nevertheless, the active Formation Period is not a direct measurement of osteoblast lifespan and the increase observed could be due to a prolonged osteoblast precursor recruitment to individual bone remodeling units, resulting in a greater total number of osteoblasts, with no alteration to individual cellular lifespan. Thus, in this study we evaluated also osteocytic apoptosis to confirm a direct action of Ris in prolonging cell lifespan. We found that Ris prevents osteocyte apoptosis in GC-treated rats, according to previous findings by Plotkin *et al. *[12]. Even if the result confirms a positive effect of Ris on cell lifespan, this effect can not completely explain our histomorphometric data that showed an increased wall thickness, the end product of osteoblastic activity, also with respect to controls.

Accordingly, it has been suggested that bisphosphonate action on bone formation may contribute to the continuous positive balance leading to a more complete mineralization of bone tissue that was highlighted also in some clinical studies [1,6,36,38]. It is still unclear how bisphosphonates could stimulate directly bone formation, even if many metabolic pathways have been suggested: the stimulation of b-FGF [10] and of Bone Morphogenetic Protein -2 (BMP-2) [17], the upregulation of a cascade of osteoblast-related genes including *BMP-2, cbfa-1, type 1 collagen and Bone Sialo-Proteins (BSP) *[18], the influence on OPG/RANKL system [2]. In addition, earlier co-culture studies have shown that the presence of osteoblastic lineage cells is required also for a complete anti-resorptive effect of bisphosphonates, suggesting that these drugs inhibit osteoclast activity both directly, interfering with mevalonate pathway, but also indirectly trough osteoblast activation [1,41]. All these findings strengthen the concept that osteoblastic lineage is another important target of aminobisphosphonates.

Among all these different mechanisms of osteoblast activation, it is known that COX-2 and endogenous prostaglandins regulate bone formation and osteoblast differentiation in bone marrow stem cells (BMSCs) [42-44]. On the contrary, COX-2 disruption in BMSC cultures decreased osteoblastogenesis, and general cell growth and inhibit or delay fracture healing [45-47].

Since COX-2 are also involved in the early stage of bone marrow stem cell differentiation toward osteoblastic lineage [42], we may suppose that bisphosphonates influence osteoblastic lineage also by promoting osteoblast differentiation and viability at least in part through COX-2 pathway. This hypothesis is in agreement with recent data that confirm COX-2 as an important target of drugs used in metabolic bone diseases, such us strontium ranelate [30].

On the other hand, administration of a drug that inhibits COX-2 activity, such as non steroidal anti-inflammatory drugs (NSAIDs), seems to be associated with an inhibition of fracture healing and reduction of bone mineral density [29], even if it is unlikely that NSAIDs are able to block continuously all PG activity at the local level *in vivo*, since PGs work in an autocrine/paracrine manner and NSAIDs are generally given for short periods.

According to these findings, our results show an increased expression of *COX-2 *in rat osteocytes treated with Ris and suggest that this pathway could be involved in the modulation of bone metabolism induced by aminobisphosphonates. As already mentioned, we found that Ris treatment prolongs osteocyte lifespan, reducing their apoptosis induced by GC. Considering that COX-2 expression and PGs increase have been associated with higher resistance to apoptosis and tumour promotion in tissues other than bone [47], the reduced apoptosis and the higher expression of COX-2 could be strictly linked.

Moreover, Ris could have a potent influence on osteoblast precursors. This is a very intriguing aspect that suggests a positive effect of bisphosphonates both on the lifespan of osteoblastic lineage and on the recruitment of new cells inducing osteogenic differentiation of mesenchimal precursors. To confirm this hypothesis, we evaluated the effect of Ris at different concentrations on *COX-2 *as well as on *b-ALP *gene expression *in vitro*.

Alkaline phosphatase (ALP), a glycoprotein attached to the outer cell membrane by a glycosylphosphatidylinositol (GPI) anchor [48], exists as several isoenzymes and many isoforms present in tissues and serum. The *ALP *gene at locus 2q34eq37 encodes the human intestinal isoenzyme of ALP whereas at locus 1p36ep34 encodes the human tissue-nonspecific ALP isoforms derived from bone, liver, and kidney [49]. In this study, we analyzed the RNA expression related to locus 1p36ep34, so in the bone marrow environment our results are referred specifically to osteogenic lineage. This aspect is very important because, in this context, b-ALP may be considered a specific differentiation marker of osteoblastic precursors. The concomitant increased expression of *COX-2 *and *b-ALP *genes after treatment with Ris suggests a direct effect on osteogenic differentiation of mesenchymal precursors. This direct effect on osteoblastic lineage has been recently suggested also in another study *in vitro*, in which zoledronate induced sustained commitment of bone marrow derived mesenchymal stem cells for osteogenic differentiation [8]. To confirm the direct relationship between *COX-2 *increase and osteoblastic activity, we co-treated cells with a specific COX-2 inhibitor (NS-398), able to decrease the mRNA *COX-2 *level [50], obtaining a concomitant significant decrease of *b-ALP *and *COX-2 *gene expression. However, the decreased *bALP *expression induced by NS-398 could be due also to the decreased viability of *bALP*-expressing cells, in addition to the effect on reduced osteogenic differentiation of mesenchymal precursors.

In addition, we evaluated Ris effects on osteocyte cells *in vitro*, and we observed similar results obtained on osteoblasts. This implies that, in addition to an increased osteogenic recruitment, Ris is able to prolong the lifespan of osteogenic lineage mature cells. Also in this case, *COX2 *gene was involved as suggested by *b-ALP *gene expression level in cells treated with Ris alone or in combination with NS-398 inhibitor. The ability of Ris to prevent negative GC effects observed on osteogenic precursors was confirmed also in MLO-Y4 osteocyte cells in agreement with our results *in vivo*.

These findings are of clinical relevance because they suggest a new mechanism through which bisphosphonates could exert their anabolic action on bone. The final result is a positive balance in bone turnover, characterized by an increased number of active osteoblastic/osteocytic cells that have an important relevance in presence of bone diseases affecting osteoblastic lineage such as glucocorticoid-induced osteoporosis. In addition, this is the first study showing a direct effect of Ris on osteocytes through the upregulation of *COX-2 *gene expression.

## Conclusions

In this study, we confirmed that osteocytes could be stimulated by aminobisphosphonates. Considering the role of osteocytes in maintaining the bone matrix network and in regulating bone metabolism for correct bone homeostasis, the effect of aminobisphosphonates on these cells is very interesting. However, further studies are needed to elucidate the effective action of drugs on these cells.

Moreover, COX-2 expression is significantly increased in osteocytes and in bone marrow cells after Ris treatment. These results suggest COX-2 as an important target of Ris and support the hypothesis that aminobisphosphonates may have an anabolic effect on bone by increasing both the lifespan and the number of active osteogenic cells.

## Abbreviations

α-MEM: α-minimum essential medium; ACTB: b-actin; AEC: 3-amino-9-ethyl-carbazole; B-ALP: bone alkaline phosphatase; BMD: bone mineral density; BMP-2: bone morphogenetic protein-2; BMSCS: bone marrow stem cells; BMU: basic multicellular unit; BPS: bisphosphonates; BSP: bone sialo-proteins; COX: cyclooxygenase; GC: glucorticoid; GPI: glycosylphosphatidylinositol; HIS: immunohistochemical score; NSAIDS: nonsteroidal anti-inflammatory drugs; PBS: phosphate-buffered saline; PGS: prostaglandins; RIS: risedronate; TUNEL: Transferase-Biotin-dUTP nick end labeling.

## Competing interests

The authors declare that they have no competing interests.

## Authors' contributions

MTV, SG and LDC were responsible for the study design. MTV, FB and SS performed animal experiments. LD and MZ performed *in vitro *experiments. LDC performed histomorphometric and statistical analyses. MTV, EO, VLC and GR were responsible for manuscript preparation.
